# Tailored Recruitment Strategies to Improve Research Study Engagement and Enrollment

**DOI:** 10.1093/geroni/igaf122.583

**Published:** 2025-12-31

**Authors:** Ruth Tappen, Joshua Chodosh

## Abstract

Successful recruitment of a sufficient number of older adults is a critical but challenging step in most research studies. In this symposium, experienced researchers will share the strategies they adopted to improve the results of their recruitment efforts. Dr. Jackson will report on the addition of a social media campaign to the more traditional community-based recruitment efforts in a large study on driving and dementia. A comparison of the sociodemographic characteristics of enrolled participants recruited via social media vs. those obtained through more traditional means provides some interesting insights. Ms. Villar reports the results of a qualitative analysis of the failure of the social media campaign to attract Spanish-speaking older adults, identifying adaptations that go beyond simple English-to-Spanish translation that should improve the response. Dr. Boon found the recruitment of older individuals with cancer and older individuals with AD achievable, but not the recruitment of those with both cancer and AD, for which an entirely different strategy was needed. Finally, Dr. Morgan reports a new, carefully constructed model to improve communication with persons with dementia that facilitates both recruitment and participation in study activities. Two of the themes that run through these papers are knowing the population of interest well and the importance of tailoring recruitment strategies to that population’s unique characteristics, values, and preferences. Our discussant, Dr. Chodosh, will elaborate on these and additional themes. Nursing Care of Older Adults Interest Group Sponsored Symposium

